# Artificial Intelligence-Based Diagnosis of Gastric Mesenchymal Tumors Using Digital Endosonography Image Analysis

**DOI:** 10.3390/jcm13133725

**Published:** 2024-06-26

**Authors:** Dong Chan Joo, Gwang Ha Kim, Moon Won Lee, Bong Eun Lee, Ji Woo Kim, Kwang Baek Kim

**Affiliations:** 1 Department of Internal Medicine, Pusan National University School of Medicine and Biomedical Research Institute, Pusan National University Hospital, Busan 49241, Republic of Korea; asclllepios@gmail.com (D.C.J.); neofaceoff@hanmail.net (M.W.L.); bongsul@hanmail.net (B.E.L.); 2 Department of Convergence Medical Sciences, The Graduate School Pusan National University, Busan 46241, Republic of Korea; kjiwoo77771@gmail.com; 3 Department of Computer Engineering, Silla University, Busan 46958, Republic of Korea; gbkim@silla.ac.kr

**Keywords:** stomach, endoscopic ultrasonography, mesenchymal tumor, artificial intelligence

## Abstract

**Background/Objectives:** Artificial intelligence (AI)-assisted endoscopic ultrasonography (EUS) diagnostic tools have shown excellent performance in diagnosing gastric mesenchymal tumors. This study aimed to assess whether incorporating clinical and endoscopic factors into AI-assisted EUS classification models based on digital image analysis could improve the diagnostic performance of AI-assisted EUS diagnostic tools. **Methods:** We retrospectively analyzed the data of 464 patients who underwent both EUS and surgical resection of gastric mesenchymal tumors, including 294 gastrointestinal stromal tumors (GISTs), 52 leiomyomas, and 41 schwannomas. AI-assisted classification models for GISTs and non-GIST tumors were developed utilizing clinical and endoscopic factors and digital EUS image analysis. **Results:** Regarding the baseline EUS classification models, the area under the receiver operating characteristic (AUC) values of the logistic regression, decision tree, random forest, K-nearest neighbor (KNN), and support vector machine (SVM) models were 0.805, 0.673, 0.781, 0.740, and 0.791, respectively. Using the new classification models incorporating clinical and endoscopic factors into the baseline classification models, the AUC values of the logistic regression, decision tree, random forest, KNN, and SVM models increased to 0.853, 0.715, 0.896, 0.825, and 0.794, respectively. In particular, the random forest and KNN models exhibited significant improvement in performance in Delong’s test (both *p* < 0.001). **Conclusion:** The diagnostic performance of the AI-assisted EUS classification models improved when clinical and endoscopic factors were incorporated. Our results provided direction for developing new AI-assisted EUS models for gastric mesenchymal tumors.

## 1. Introduction

Subepithelial tumors (SETs) are frequently coincidentally discovered during endoscopic examinations of the gastrointestinal (GI) tract. The frequency of upper GI SETs is reported to be 1.6−1.9%; approximately two-thirds of upper GI SETs are present in the stomach [1,2]. Among gastric SETs, gastric mesenchymal tumors originating from the muscularis propria are of major concern in clinical practice. Gastric mesenchymal tumors include benign tumors such as leiomyomas or schwannomas and malignant tumors such as gastrointestinal stromal tumors (GIST) [3,4,5]. Therefore, differentiating GISTs from nonmalignant tumors, including leiomyomas and schwannomas, is important for managing gastric mesenchymal tumors [3].

Various modalities are used to diagnose gastric SETs. White-light endoscopy enables assessment of the size, location, and macroscopic appearance of the tumor via direct visualization. However, white-light endoscopy cannot provide sufficient information regarding the originating layer and inner characteristics of the tumor. Furthermore, endoscopic forceps biopsy is not always feasible due to the subepithelial nature of such tumors, particularly those located in the muscularis propria [6,7,8,9]. Computed tomography (CT) is commonly used to diagnose gastric SETs. CT is beneficial for detecting the extragastric invasion and metastasis of tumors. However, CT cannot detect small gastric SETs, particularly when the tumor size is less than 1 cm [6,10,11]. Endoscopic ultrasonography (EUS) provides comprehensive information on the tumor, including the originating layer, size, shape, internal echo pattern, and heterogeneity of the tumor and the presence or absence of internal cystic changes and calcification. Consequently, EUS is regarded as the most valuable diagnostic tool for evaluating gastric SETs. However, the diagnosis of SETs by EUS is operator-dependent; thus, interobserver variability and sometimes intraobserver variability are major limitations [12,13,14,15,16]. Accordingly, tissue acquisition using EUS guide, such as EUS-guided fine-needle aspiration (EUS-FNA) and EUS-guided fine-needle biopsy (EUS-FNB), is employed to establish a definitive histopathological diagnosis in patients with SETs. Recent meta-analyses have shown that EUS-FNB could provide satisfactory levels of sample adequacy and diagnostic accuracy (94.9% and 87.9%, respectively) [17]. However, EUS-FNA/FNB is not available in all hospitals and requires advanced technical skills and experience. Furthermore, EUS-FNA/FNB is an invasive procedure with procedure-related adverse events such as bleeding or infection [18]. Additionally, its diagnostic accuracy rate for small SETs is not as satisfactory as that for large SETs [10,18,19,20].

Images in digital form are made up of pixels, which are the fundamental building blocks of a flat image. Our prior study showed that analyzing digital EUS images can offer unbiased data to distinguish between GISTs and non-GIST tumors in gastric mesenchymal tumors; it can reduce variations in interpretation among different observers [21]. The scoring system, which involves digital image analysis and clinical characteristics, demonstrates high sensitivity and specificity for predicting GISTs in gastric mesenchymal tumors [4]. Artificial intelligence (AI) technology has recently been applied to the medical field, particularly with deep learning techniques involving convolutional neural networks [18,22,23]. This technology is increasingly being used in endoscopic diagnostics, particularly for identifying and evaluating esophageal cancer, gastric cancer, and colorectal polyps, as well as for assessing pancreatic lesions [24,25,26,27]. AI-assisted EUS diagnostic tools have been introduced in gastric SETs to overcome the limitations of the current diagnostic modalities [18,28]. They have demonstrated excellent diagnostic performance in diagnosing GI SETs in a recent meta-analysis [16]. However, the current AI-assisted EUS diagnostic tools also have limitations. Previous studies have shown that the diagnostic accuracy of AI-assisted EUS diagnostic tools for detecting SETs decreases as tumor size decreases [29]. Considering these studies included EUS image data alone for AI-assisted EUS diagnostic tools, this limitation may arise from the fact that EUS characteristics of GISTs, such as marginal irregularity and cystic changes, are often not clearly discernible in EUS images of small-sized GISTs. According to previous studies, gastric GISTs are associated with clinical and endoscopic characteristics such as age, sex, tumor location, and ulceration [4,14,30]. Thus, AI-assisted EUS diagnostic tools, including clinical and endoscopic factors and digital EUS image analysis, could improve diagnostic performance for gastric mesenchymal tumors. Therefore, we aimed to evaluate whether incorporating clinical and endoscopic factors into an AI-assisted EUS diagnostic tool based on digital image analysis could improve diagnostic performance compared with AI-assisted EUS diagnostic tools using EUS images alone.

## 2. Materials and Methods

### 2.1. Study Population

The information of 464 patients who received EUS and surgical resection of gastric SETs at Pusan National University Hospital from March 2009 to August 2021 was retrospectively reviewed. Among them, 294 patients with GISTs, 52 with leiomyomas, and 41 with schwannomas were included in this study. Based on the potential for malignancy, these patients were grouped into GIST and non-GIST groups. This study was reviewed and received approval by the Institutional Review Board of Pusan National University Hospital (IRB number: 2405-007-139).

### 2.2. EUS

EUS was performed using a radial-scanning echoendoscope (GF-UM2000 and GF-UE260-AL5; Olympus, Tokyo, Japan) at 7.5 MHz. All tests were conducted while the patient was under intravenous conscious sedation using midazolam with or without propofol. After injecting 200–500 mL of deaerated water into the stomach, tumors were examined. A minimum of 10 EUS images were captured for every lesion and stored digitally in Windows bitmap format. A single experienced endosonographer (G.H.K.) reviewed the EUS images without knowing about the final diagnosis. A single EUS image of the highest quality was selected for each lesion to undergo further digital image analysis on a standard desktop computer.

During EUS, endoscopic characteristics, such as the presence or absence of ulceration and longitudinal and transverse tumor location, were recorded. The longitudinal tumor location was classified as cardia, upper third (fundus and upper body), middle third (midbody and angle), and lower third (antrum and pylorus). Cardia was defined as the center of the tumor located within 2 cm distal to the esophagogastric junction [31]. The transverse tumor location was classified as the anterior wall, lesser curvature, posterior wall, and greater curvature. 

### 2.3. Histopathology

The tumors were immunohistochemically classified into GIST, leiomyoma, or schwannoma [4,32]. A GIST was characterized as a tumor positive for c-kit, DOG-1, or CD34; leiomyoma was identified as a tumor positive for desmin and negative for c-kit (CD117); and schwannoma was defined as a tumor positive for S-100 and negative for c-kit. Histopathologic diagnosis was made by two pathologists specializing in gastroenterology. When their diagnoses did not coincide, a consensus diagnosis was made using a multiheaded microscope.

### 2.4. Digital Image Analysis

**Figure 1:**
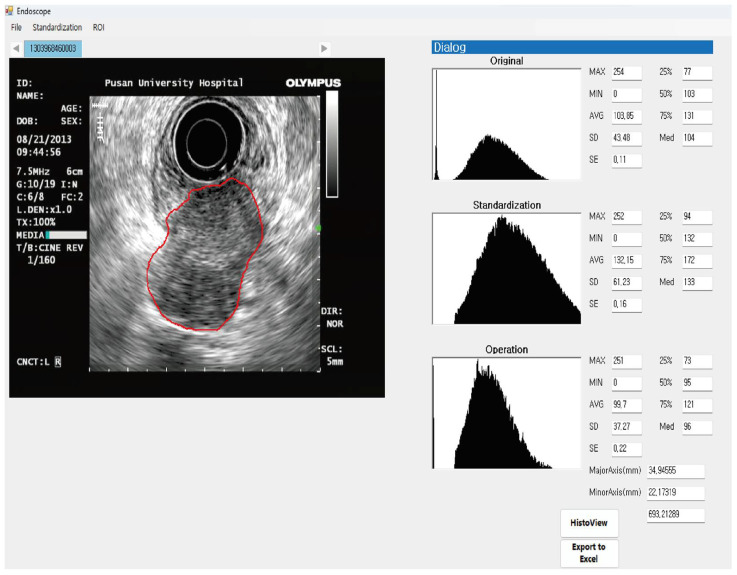
An illustration of analyzing digital endoscopic ultrasonography (EUS) images. A region of interest (ROI) is selected from the standardized image for tumor analysis. The final results for the ROI are shown on the right-hand side of the histogram.

EUS can exhibit varying image characteristics depending on different contrasts during an actual examination. Therefore, the standardization process and extraction of brightness information from the EUS images were performed as described in our previous studies [4,21]. The process of standardization employed the brightness values from the anechoic center and outer hyperechoic rim of the echoendoscope. In the standardized EUS images, an experienced endosonographer (G.H.K.) identified the region of interest (ROI) for a detailed tumor analysis. This method yielded various brightness information, including the mean brightness value (T_mean_), which reflected the tumor’s echogenicity, and the standard deviation (T_SD_) of the brightness value, indicative of the heterogeneity of echogenicity in the tumor (Figure 1) [4].

### 2.5. AI Model for Differentiating GISTs from Non-GIST Tumors

Given the relatively small size of the dataset, GIST and non-GIST tumor classification models were trained and tested using simple algorithms like logistic regression, decision tree, random forest, K-nearest neighbor (KNN), and support vector machine (SVM). Logistic regression, one of the most commonly used algorithms in medical data analysis, categorizes data into two groups by employing a probability model that predicts the event likelihood through a linear combination of independent variables [33,34]. The decision tree algorithm classifies the data based on specific criteria, with the risk of overfitting if built without constraints. To prevent the overfitting problem, pruning techniques, such as setting maximum tree depth or limiting terminal nodes, are applied [35]. The random forest algorithm improves prediction accuracy by randomly selecting features and aggregating predictions from multiple decision trees. This ensemble method combines several weak classifiers to form a stronger, more accurate classifier [36,37]. The KNN algorithm determines a sample’s classification by the prevalent class among its closest neighbors, with ‘K’ setting the count of neighbors to examine [38]. The SVM algorithm is a binary linear classification technique that works by finding the optimal hyperplane to separate different groups [39].

**Figure 2:**
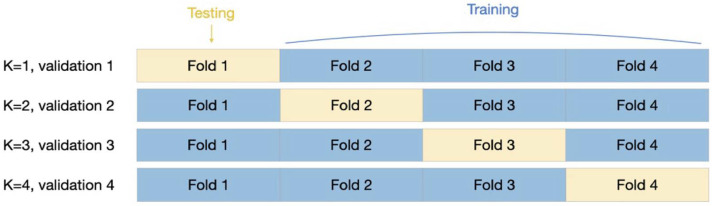
K-fold cross-validation. In this approach, the training dataset was divided into K smaller subsets. The model was trained on K-1 of these subsets, while the remaining subset was used as the test set for evaluating performance. This process was repeated K times, with each of the K subsets serving as the test set once.

The data collected in this study exhibited an imbalance in sample sizes between the two groups (294 and 93 patients in the GIST and non-GIST groups, respectively). To address the issue of overfitting owing to an imbalanced dataset, a stratified K-fold cross-validation technique was utilized. This process involves dividing the original data into folds, using each fold in turn for testing and the rest for training (Figure 2). Stratified K-fold cross-validation is particularly effective in handling imbalanced datasets in disease classification models because it ensures that each fold maintains the same proportion in each group as the original dataset. Therefore, this technique enhances the reliability of evaluation by ensuring that models are not skewed towards the more prevalent group of the original dataset [36].

### 2.6. Statistical Analysis

All continuous variables, including age and tumor size, are expressed as mean ± standard deviation (SD). A Student’s *t*-test was applied to evaluate the difference in continuous variables between the GIST and non-GIST groups. Categorical variables were analyzed using the chi-squared test or Fisher’s exact test. The performance of each classification model for differentiating GISTs from non-GIST tumors was assessed using a receiver operating characteristic (ROC) curve and Delong’s test. Statistical analyses were conducted using IBM SPSS version 27.0 for Windows (IBM Co., Armonk, NY, USA), with a *p*-value of less than 0.05 considered statistically significant.

## 3. Results

### 3.1. Baseline Clinical and Endoscopic Characteristics of Patients with a Gastric Mesenchymal Tumor

The baseline characteristics of 387 patients with gastric mesenchymal tumors are presented in Table 1. The patients included 159 men and 228 women, with a mean age of 58.2 years. The mean size of the gastric mesenchymal tumors was 3.1 cm. Regarding longitudinal tumor location, 37 tumors were located in the gastric cardia, 190 in the upper third of the stomach, 134 in the middle third, and 26 in the lower third. Regarding the transverse tumor location, 64 were located at the anterior wall, 57 at the lesser curvature, 119 at the posterior wall, and 147 at the greater curvature. Forty-five tumors had ulceration on their surface. 

### 3.2. Univariate Analysis of Factors Predicting GISTs

Patients in the GIST group were significantly older compared with those in the non-GIST group (61.9 ± 10.7 years vs. 52.1 ± 12.2 years, *p* < 0.001). GISTs were more frequently located in the upper third, and non-GIST tumors were more frequently located in the cardia (*p* < 0.001). There was no difference in sex, tumor size, transverse tumor location, and presence of ulceration between the two groups (Table 2). After poststandardized image analysis, both T_mean_ and T_SD_ were successfully calculated in all EUS images. The GIST group exhibited a significantly higher T_mean_ compared with the non-GIST group (63.2 ± 18.8 vs. 42.7 ± 19.2, *p* < 0.001). The T_SD_ was also significantly higher in the GIST group than that in the non-GIST group (19.3 ± 5.1 vs. 16.2 ± 5.2, *p* < 0.001).

### 3.3. Developing AI Models for Differentiating GISTs from Non-GIST Tumors

In previous AI studies, many classification models for gastric mesenchymal tumors were created using EUS images alone. Therefore, baseline classification models were developed based on results using digital EUS image analysis, including T_mean_ and T_SD_ of the tumor. In contrast, new classification models for gastric mesenchymal tumors have been developed by adding clinical and endoscopic factors to digital EUS image analysis. Among the clinical and endoscopic factors, age and longitudinal tumor location, which were significant factors for predicting GISTs using the univariate analysis, were included. GIST and non-GIST classification models were trained and tested using logistic regression, decision tree, random forest, KNN, and SVM algorithms with K-fold cross-validation.

### 3.4. Classification Performance of AI Models

**Figure 3:**
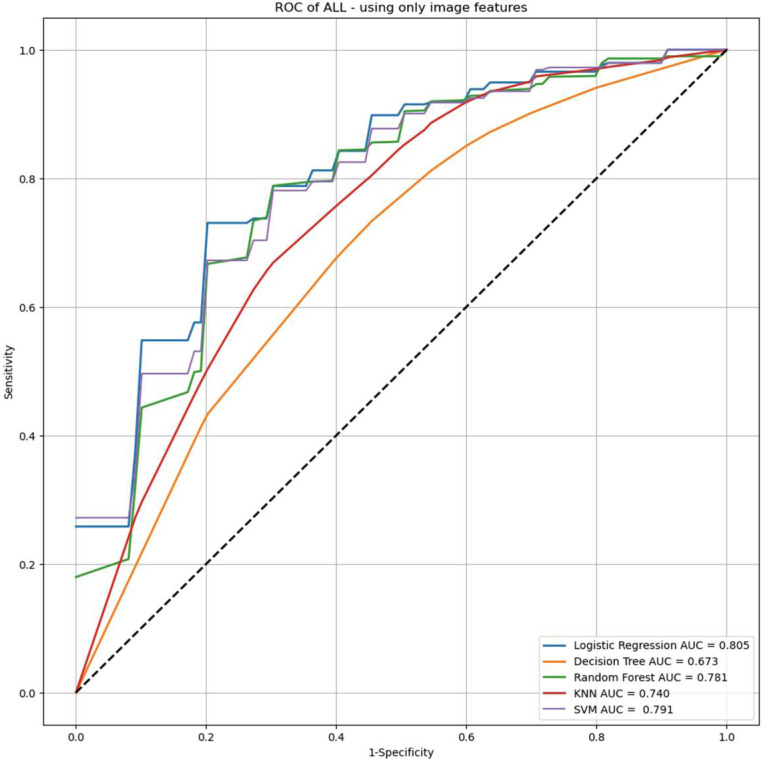
Receiver operating characteristic curves of baseline artificial intelligence (AI)-assisted EUS classification models, including digital EUS image analysis alone.

**Figure 4:**
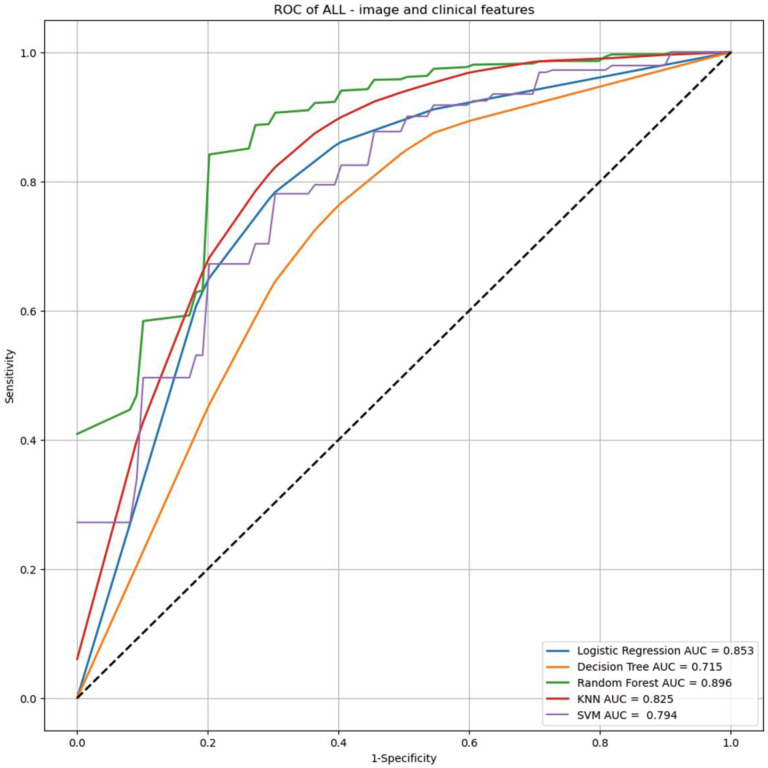
Receiver operating characteristic curves of new AI-assisted EUS classification models incorporating clinical and endoscopic factors (age and tumor location) to baseline AI-assisted classification models.

The detailed diagnostic performance of each AI model for differentiating GISTs from non-GIST tumors is summarized in Table 3. Regarding the baseline classification models, the area under the ROC curve (AUC) values of the logistic regression, decision tree, random forest, KNN, and SVM models were 0.805 (95% confidence interval [CI], 0.753–0.855), 0.673 (95% CI, 0.580–0.710), 0.781 (95% CI, 0.723–0.836), 0.740 (95% CI, 0.666–0.795), and 0.791 (95% CI, 0.666–0.795), respectively (Figure 3). In the new classification models incorporating clinical and endoscopic factors (age and tumor location) into the baseline classification models, the AUC values of the logistic regression, decision tree, random forest, KNN, and SVM models increased to 0.853 (95% CI, 0.799–0.892), 0.715 (95% CI, 0.668–0.772), 0.896 (95% CI, 0.825–0.919), 0.825 (95% CI, 0.766–0.898), and 0.794 (95% CI, 0.732–0.856), respectively (Figure 4). These differences in the logistic regression, decision tree, and SVM models did not reach statistical significance in Delong’s test (*p* = 0.057, *p* = 0.232, and *p* = 0.904, respectively). However, the random forest and KNN models showed significant improvement in performance in Delong’s test (both *p* < 0.001).

## 4. Discussion

In this study, we attempted to develop AI-assisted models for differentiating GISTs from non-GIST tumors based on digital EUS image analysis of gastric mesenchymal tumors. When only digital EUS image analysis was included, the AUC values of the logistic regression, decision tree, random forest, KNN, and SVM models were 0.805, 0.673, 0.781, 0.740, and 0.791, respectively. The AUC values of all five models improved numerically when the clinical and endoscopic factors were incorporated. The AUC values of the random forest and KNN models were significantly improved to 0.896 and 0.825, respectively. To our best knowledge, this is the first study to report AI-assisted models that could predict GISTs using clinical and endoscopic characteristics and digital EUS image analysis.

In a recent meta-analysis involving eight retrospective studies including 2,355 patients, AI-assisted EUS models demonstrated a sensitivity of 92% (95% CI, 89–95%), specificity of 80% (95% CI, 75–85%), and AUC value of 0.949 for diagnosing GISTs [16]. However, experienced endoscopists demonstrated a sensitivity of 72% (95% CI, 67–76%), specificity of 70% (95% CI, 64–76%), and AUC value of 0.777 for diagnosing GISTs. Regarding the performance for differentiating GISTs from leiomyomas, the AI-assisted EUS models demonstrated a sensitivity of 93% (95% CI, 88–97%), specificity of 90% (95% CI, 88–95%), and AUC value of 0.966. Experienced endoscopists demonstrated a sensitivity of 73% (95% CI, 65–80%), a specificity of 75% (95% CI, 65–84%), and an AUC value of 0.819. These results indicate a substantial improvement in diagnosing gastric mesenchymal tumors with the incorporation of AI technologies. However, the diagnostic performance of AI-assisted EUS models for detecting SETs <2 cm in size was not high compared with that of SETs >2 cm in size [29], which might be because EUS characteristics of GISTs, such as marginal irregularity and cystic changes, are not often clearly discernible in small-sized GISTs [40]. This issue could be explained by previous AI-assisted EUS models focusing solely on the EUS images without considering clinical and endoscopic factors.

Gastric SETs have several clinical characteristics, such as age and tumor location, according to histopathology [20,40,41,42,43]. Old age and location in the gastric body and fundus are associated with GISTs, and female sex and location in the gastric cardia are associated with leiomyomas. Similarly, in this study, patients in the GIST group were markedly more advanced in age compared with those in the non-GIST group, with an average age of 61.9 years versus 52.1 years. Tumor location in the upper third of the stomach was associated with GISTs, whereas tumor location in the gastric cardia was associated with non-GIST tumors. In digital EUS image analysis, T_mean_ and T_SD_ were markedly elevated in the GIST group compared with the non-GIST group, consistent with the results of our previous studies [4,21]. 

Recently, Yu et al. developed AI diagnostic models to identify patients who were diagnosed with COVID-19 using CT findings combined with laboratory tests [44]. In the study, integrating CT results with laboratory findings significantly enhanced the diagnostic performance in all AI diagnostic models. These findings suggest that an AI-assisted diagnostic model incorporating various data types with imaging results can enhance diagnostic performance. Similarly, to ascertain which clinical and endoscopic characteristics should be included in the development process to improve the diagnostic performance of AI-assisted EUS models, we integrated patient age and tumor location into new AI-assisted EUS classification models for differentiating GISTs from non-GIST tumors based on the results of univariate analysis. The AUC values of the logistic regression, decision tree, random forest, KNN, and SVM models improved from 0.805, 0.673, 0.781, 0.740, and 0.791, respectively, to 0.853, 0.715, 0.896, 0.825, and 0.794. Particularly, the random forest and KNN models exhibited significant improvements in classification performance.

Compared with other studies on AI-assisted EUS models for gastric SETs, our results exhibited a relatively low AUC value (maximum 0.896 in the random forest model) for differentiating GISTs from non-GIST tumors. These results may be attributed to the relatively small number of tumors included in the present study and use of limited data (only T_mean_ and T_SD_ in digital EUS image analysis) based on digital EUS image analysis. However, as previously mentioned, this study aimed to confirm that an AI-assisted EUS models incorporating clinical and endoscopic factors could improve diagnostic performance compared with an AI-assisted EUS models using EUS images alone. We found that incorporating clinical and endoscopic factors into AI-assisted EUS models could improve the classification performance for gastric mesenchymal tumors. Our findings highlight the importance of incorporating clinical and endoscopic features in developing future AI-assisted EUS models. We expect the relatively low AUC values in this study to increase as we plan to use the EUS images themselves rather than limited data in digital EUS image analysis in subsequent studies based on the results of this study.

This study has some limitations. First, this was a single-center, retrospective study. Therefore, the sample size was relatively small; there might have been a potential bias in the retrospective review and selection of EUS images. Second, GISTs are malignant tumors that often require surgical resection, whereas non-GIST tumors generally do not need to be resected. Since surgery was performed based on the clinical judgment of the endoscopists, there was an imbalance in the composition of the study groups between those diagnosed with GISTs and non-GIST tumors. Third, we included only EUS images taken at 7.5 MHz to lessen differences among the images according to the use of different frequencies. However, the EUS settings including gain and contrast, as well as different types of echoendoscopes and EUS systems, varied in each case. Thus, we standardized the EUS images based on the brightness values from the anechoic center and the outer hyperechoic rim of the echoendoscope. However, this standardization process could not completely overcome the limitations of this retrospective study. Further large-scale, prospective, multicenter studies using consistent EUS settings are needed in future. Fourth, since this study was a preliminary study that aimed to confirm the benefit of integrating clinical and endoscopic features in AI-assisted EUS diagnostic model, we did not include the interobserver variability. We plan to include the difference in the diagnostic accuracy between endoscopists and AI models as well as the interobserver variability in further studies on AI-assisted EUS diagnostic models.

## 5. Conclusions

The diagnostic performance of the AI-assisted EUS classification models improved when clinical and endoscopic characteristics were incorporated. Our results could provide direction for developing new AI-assisted EUS models for gastric mesenchymal tumors.

## Figures and Tables

**Table 1:** Baseline characteristics of the study population.

Characteristics	Number
Age (years, mean ± SD)	58.2 ± 11.7
Sex, n (%)	
Male	159 (41.1)
Female	228 (58.9)
Size (cm, mean ± SD)	3.1 ± 1.9
Longitudinal location, n (%)	
Cardia	37 (9.6)
Upper third	190 (49.1)
Middle third	134 (34.6)
Lower third	26 (6.7)
Transverse location, n (%)	
Anterior	64 (16.5)
Lesser curvature	57 (14.7)
Posterior	119 (30.8)
Greater curvature	147 (38.0)
Ulceration, n (%)	
Absent	342 (88.4)
Present	45 (11.6)

SD, standard deviation.

**Table 2:** Comparison of clinical and endoscopic characteristics and digital EUS image analysis between gastrointestinal stromal tumor (GIST) and non-GIST groups.

Features	Non-GIST Group (n = 93)	GIST Group (n = 294)	*p*-Value
Age (years, mean ± SD)	52.1 ± 12.2	61.9 ± 10.7	<0.001
Sex, n (%)			0.309
Male	34 (36.6)	125 (42.5)	
Female	59 (63.4)	169 (57.5)	
Size (cm, mean ± SD)	3.2 ± 1.9	3.1 ± 1.9	0.671
Longitudinal location, n (%)			<0.001
Cardia	32 (34.4)	5 (1.7)	
Upper third	22 (23.7)	168 (57.1)	
Middle third	35 (37.6)	99 (33.7)	
Lower third	4 (4.3)	22 (5.7)	
Transverse location, n (%)			0.231
Anterior wall	12 (12.9)	52 (17.7)	
Lesser curvature	8 (8.6)	49 (16.7)	
Posterior wall	38 (40.9)	81 (27.6)	
Greater curvature	35 (37.6)	112 (38.1)	
Ulceration, n (%)			0.763
Absent	83 (89.2)	259 (88.1)	
Present	10 (10.8)	35 (11.9)	
T_mean_ (mean ± SD)	42.7 ± 19.2	63.2 ± 18.8	<0.001
T_SD_ (mean ± SD)	16.2 ± 5.2	19.3 ± 5.1	<0.001

GIST, gastrointestinal stromal tumor; SD, standard deviation.

**Table 3:** Diagnostic performance of baseline model and new model for differentiating GISTs from non-GIST tumors.

		Diagnostic Performance				
		AUC (95% CI)	Sensitivity, % (95% CI)	Specificity, % (95% CI)	PPV, % (95% CI)	NPV, % (95% CI)
Baseline model (only EUS images)	Logistic regression	0.805 (0.753–0.855)	65.6 (62.5–68.5)	90.9 (78.5–92.0)	81.8 (80.3–85.3)	66.3 (61.6–71.1)
	Decision tree	0.673 (0.580–0.710)	71.9 (67.5–77.1)	45.5 (34.6–58.1)	79.3 (76.9–83.5)	63.1 (60.0–68.0)
	Random forest	0.781 (0.723–0.836)	78.1 (74.9–84.3)	81.8 (64.4–89.9)	78.5 (70.2–84.9)	64.0 (50.7–77.4)
	KNN	0.740 (0.666–0.795)	78.8 (69.6–79.4)	84.3 (72.3–85.4)	85.5 (79.3–88.8)	62.6 (52.9–64.3)
	SVM	0.791 (0.666–0.795)	93.9 (79.9–94.9)	74.9 (66.2–83.7)	75.1 (73.0–80.3)	52.9 (36.0–59.9)
New model (integrating clinical and endoscopic factors)	Logistic regression	0.853 (0.799–0.892)	87.5 (83.2–91.7)	90.9 (80.4–95.1)	88.2 (82.5–93.6)	85.0 (80.4–89.7)
	Decision tree	0.715 (0.668–0.772)	93.8 (88.9–96.2)	63.6 (62.9–69.2)	88.4 (85.1–90.7)	77.0 (76.2–83.0)
	Random forest	0.896 (0.825–0.919)	93.8 (87.8–94.5)	81.8 (70.6–86.7)	91.4 (85.0–96.0)	75.1 (70.3–79.9)
	KNN	0.825 (0.766–0.898)	93.9 (85.5–96.4)	81.1 (74.9–82.3)	93.2 (84.4–96.2)	80.6 (69.3–83.0)
	SVM	0.794 (0.732–0.856)	93.9 (85.0–96.6)	80.5 (69.9–91.0)	79.5 (77.4–81.8)	52.2 (70.0–85.3)

AUC, area under the ROC curve. CI, confidence interval. PPV, positive predictive value. NPV, negative predictive value. EUS, endoscopic ultrasonography. KNN, K-nearest neighbor. SVM, support vector machine.

## Data Availability

The data that support the findings of this study can be obtained from the corresponding author upon reasonable request.
